# Immune profiling of critically ill patients with acute kidney injury during the first week after various types of injuries: the REALAKI study

**DOI:** 10.1186/s13054-024-04998-w

**Published:** 2024-07-08

**Authors:** Frank Bidar, Louis Peillon, Maxime Bodinier, Fabienne Venet, Guillaume Monneret, Anne-Claire Lukaszewicz, Jean-François Llitjos, Julien Textoris, Thomas Rimmelé

**Affiliations:** 1 Anesthesia and Critical Care Medicine Department, Edouard Herriot Hospital, Hospices Civils de Lyon, Lyon, France; 2 EA 7426 “Pathophysiology of Injury-Induced Immunosuppression” (Université Claude Bernard Lyon 1 - Hospices Civils de Lyon - bioMérieux), Lyon, France; 3 Immunology Laboratory, Edouard Herriot Hospital, Hospices Civils de Lyon, Lyon, France; 4 NLRP3 Inflammation and Immune Response to Sepsis Team, Centre International de Recherche in Infectiology (CIRI), Inserm U1111, CNRS, UMR5308, Ecole Normale Supérieure de Lyon, Claude Bernard University Lyon 1, Lyon, France

**Keywords:** Acute kidney injury, Burns, Immunosuppression, Inflammation, Intensive care, Sepsis, Surgery, Trauma

## Abstract

**Background:**

Acute kidney injury (AKI) is common in hospitalized patients and results in significant morbidity and mortality. The objective of the study was to explore the systemic immune response of intensive care unit patients presenting with AKI, especially the association between immune profiles and persistent AKI during the first week after admission following various types of injuries (sepsis, trauma, surgery, and burns).

**Methods:**

REALAKI is an ancillary analysis of the REAnimation Low Immune Status Marker (REALISM) cohort study, in which 359 critically ill patients were enrolled in three different intensive care units. Patients with end-stage renal disease were excluded from the REALAKI study. Clinical samples and data were collected three times after admission: at day 1 or 2 (D1-2), day 3 or 4 (D3-4) and day 5, 6 or 7 (D5-7). Immune profiles were compared between patients presenting with or without AKI. Patients with AKI at both D1-2 and D5-7 were defined as persistent AKI. A multivariable logistic regression model was performed to determine the independent association between AKI and patients’ immunological parameters.

**Results:**

Three hundred and fifty-nine patients were included in this analysis. Among them, 137 (38%) were trauma patients, 103 (29%) post-surgery patients, 95 (26%) sepsis patients, and 24 (7%) were burn patients. One hundred and thirty-nine (39%) patients presented with AKI at D1-2 and 61 (20%) at D5-7. Overall, 94% presented with persistent AKI at D5-7. Patients with AKI presented with increased pro and anti-inflammatory cytokines and altered innate and adaptive immune responses. The modifications observed in the immune profiles tended to be more pronounced with increasing KDIGO stages. In the logistic regression model, a statistically significant association was observed at D1-2 between AKI and CD10^low^CD16^low^ immature neutrophils (OR 3.03 [1.7–5.5]—*p* < 0.001). At D5-7, increased interleukin-10 (IL-10) levels and reduced ex vivo TNF-α production after LPS stimulation were significantly associated with the presence of AKI (OR 1.38 [1.12–1.71]—*p* = 0.001 and 0.51 [0.27–0.91]—*p* = 0.03, respectively). Patients who recovered from AKI between D1-2 and D5-7 compared to patients with persistent AKI at D5-7, tended to correct these alterations.

**Conclusion:**

Following various types of severe injuries, early AKI is associated with the initial inflammatory response. Presence of AKI at the end of the first week after injury is associated with injury-induced immunosuppression.

**Supplementary Information:**

The online version contains supplementary material available at 10.1186/s13054-024-04998-w.

## Background

Acute kidney injury (AKI) is common in hospitalized patients and results in significant morbidity and mortality [1]. It affects up to 75% of critically ill patients and occurs in many different clinical situations such as sepsis, trauma, surgery or burns [2]. Historically, kidney dysfunction was mainly related to hemodynamic disturbances, however many studies over the last twenty years have explored the interaction between AKI, inflammation, and the immune system. AKI is nowadays considered as a systemic syndrome and inflammation is known to play a major role in AKI pathophysiology [3]. Increased levels of inflammatory cytokines such as interleukin-6 (IL-6) are associated with the development of AKI in septic or cardiac surgery patients [4, 5]. Furthermore, the survivors among critically ill patients who presented with an AKI may develop progressive kidney disease. Elevated concentrations of inflammatory and apoptosis biomarkers in these patients are associated with non-recovery of kidney function, dialysis dependence and death [6]. Therefore, understanding the immune response of critically ill patients with AKI could help to develop immunomodulation strategies to improve kidney recovery.

In critically ill patients, the initial injury leading to intensive care unit (ICU) hospitalization is often followed by a maladaptive injury-acquired immunodeficiency syndrome. This state of “immunoparalysis” is known to increase the occurrence of healthcare associated infections (HAI) and is associated with both morbidity and mortality in ICU patients [7]. In addition, AKI itself may alter the immune system response and is recognized as an independent risk factor for sepsis [8]. A significant proportion of AKI-related mortality is not only due to the alteration of the kidney function but may also be explained by simultaneous illnesses during the hospitalization of AKI patients. Among them, sepsis is the primary cause of death in hospitalized patients and may account for more than 40% of mortality in patients with AKI [9]. This increased risk may be related to the initial inflammatory response and subsequent immunoparalysis in response to the initial injury. Both alterations induced by the initial injury and the development of AKI itself may explain the morbidity and mortality observed in AKI patients. Thus, identifying the modifications in the host immune response in patients presenting with AKI could help reduce AKI-related morbidity and mortality. Despite extensive experimental research [10], few studies have reported the dysregulation of the immune system in ICU patients with AKI over time [5, 11, 12].

AKI typically occurs early during the ICU stay and is present at ICU admission in most patients [13]. Persistent AKI defined by a sustained kidney dysfunction beyond 48–72 h from AKI onset is associated with worse outcomes [14, 15]. Therefore, the current study sought to perform an immune profiling of ICU patients presenting with AKI during the first week after admission following various types of injuries.The secondary objective was to determine the association between immune profile and persistent AKI at the end of the first week after injury.

## Methods

### Design

REALAKI is a secondary analysis of the REAnimation Low Immune Status Marker (REALISM) study, in which critically ill patients with various types of injuries (sepsis, trauma or elective surgery) were enrolled, as well as 175 healthy volunteers [16, 17]. The objective of the REALISM study was to broadly assess immune profiles in a cohort of critically ill patients during the first two months after an injury and to correlate these findings with clinical epidemiological data and outcomes.

Inclusion criteria comprised patients aged > 18 years, clinical diagnosis of sepsis as defined by the 2016 SEPSIS-3 consensus guidelines (1), severe trauma with an injury severity score > 15, surgical patients undergoing major surgeries such as esophago-gastrectomy, bladder resection with Brickers’ reconstruction, cephalic pancreaticoduodenectomy and abdominal aortic aneurysm surgery by laparotomy and severe burn patients with a total burn surface area over 30%. Patients’ demographics, comorbidities, diagnosis, severity, and clinical outcome were prospectively collected. Exclusion criteria were any of the following: presence of a preexistent condition or treatment that could interfere with patient’s immune status, pregnancy, institutionalized patients, inability to obtain informed consent. Patients presenting with end-stage renal disease prior to enrollment in the REALISM study were excluded from the present REALAKI study.

Written informed consent was obtained from each patient upon inclusion. The REALISM study protocol was approved on December 3, 2015, by the Institutional Review Board (Comité de Protection des Personnes Sud-Est II) under number 2015-42-2 and was in accordance with the 1975 Helsinki Declaration. It was also registered at ClinicalTrials.gov (NCT02638779).

### Blood sampling and data collection

Clinical samples and data were collected three times during the first week after admission: at day 1 or 2 (D1-2), day 3 or 4 (D3-4) and day 5, 6 or 7 (D5-7). Peripheral whole blood was collected at each time-point for each patient. Tubes were immediately transferred to the lab and processed within 3 h after blood sampling for flow cytometry immune phenotyping and plasma cytokine level measurements. The protocol for sampling has been described previously [17]. Immune parameters included in the study and their significance are reported in Additional table S1.

### Outcomes

AKI was defined according to the Kidney Disease Improving Global Outcomes (KDIGO) guidelines [18]. AKI was defined at D1-2, D3-4 and D5-7 as present or not at the corresponding time-point. Only creatinine values were used to define AKI as urine output values were not collected prospectively in all patients, especially when patients were discharged from the ICU and transferred to the ward. We determined a value for baseline creatinine level using a hierarchical approach in which creatinine values obtained during the year before hospitalization were given priority over in-hospital measurements obtained before ICU admission. Whenever no baseline creatinine value was available, the baseline creatinine level was estimated using the Modification of Diet in Renal Disease formula [19]. In this case, the eGFR used to estimate the baseline creatinine level was 90 mL/min/1.73m^2^. If patients had prior history of CKD, we used the most recent eGFR or creatinine value available to define the baseline value.

The definition of persistent AKI was based on the definition suggested by the Acute Disease Quality Initiative (ADQI) workgroup, which is the continuance of AKI defined by KDIGO classification, beyond 48 h from AKI onset [14]. Therefore, in the present study, patients with AKI at both D1-2 and D5-7 were defined as persistent AKI patients, although the duration could be longer than 48 h. Patients with late-onset AKI were defined as patients with AKI at D5-7 while presenting no AKI at D1-2 or D3-4.

HAI were reported prospectively during the hospital stay. Patients were screened daily for exposure to invasive devices (intubation, indwelling urinary catheter and central venous line) and occurrence of secondary infection. Information related to infections was collected, reviewed and validated by a blinded dedicated adjudication committee, composed of three physicians not involved in the recruitment of the patients with confirmation of secondary infection made according to the definition used by the European Centre for Disease Prevention and Control [20]. Clinical worsening was defined as the occurrence of death or HAI, as previously described, during the follow-up.

### Statistical analysis

Qualitative variables are presented as numbers and percentages and quantitative variables as medians and 25th/75th percentiles. Chi square or Fisher’s exact tests were used for qualitative variables assessment. Quantitative variables were compared with Mann–Whitney U test or Student t tests according to the distribution of the variables. Normality was assessed using histograms and Shapiro–Wilk test. The comparisons between immunological parameters according to the AKI subgroups were adjusted for multiple comparisons using the Bonferroni method.

A multivariable logistic regression model using a backward stepwise selection was performed to determine the independent association between AKI and patients’ immunological parameters. A first model was established to determine the association between parameters measured at D1-2 and the presence of an AKI at D1-2. Similarly, a second model was established for D5-7. Variables included in these models were immune parameters significantly associated with AKI in the univariable analysis (with *p*-value < 0.05) and clinical variables of interest namely age, sex, modified Sequential Organ Failure Assessment (SOFA) score at admission (i.e. SOFA score excluding the renal component), the renal component of the Charlson score and the type of injury. Lastly, a model was performed to determine the association between the initial pro and anti-inflammatory response measured at D1-2 and the presence of a persistent AKI at D5-7. This model was adjusted on clinical variables of interest namely age, sex, and presence of AKI at D1-2. Between-variable interactions were sought in the models; variables strongly associated with other(s) were not included in the multivariable model. Odds ratios calculated for all immune parameters were normalized to an increment from first to third quartile to allow comparisons between immune parameters.

A linear mixed model (LMM) that accounted for the interaction between time-points and the AKI status at D5-7 (recovery from AKI or persistent AKI) was performed, incorporating random effects for patients to handle intra-subject correlation of repeated measurements. To meet the assumptions of the LMM and ensure appropriate analysis, IL-6 and IL-10 data were log-transformed prior to modeling. Pairwise comparison tests were performed between groups and timepoints using estimated marginal means derived from the LMM. Adjustments for multiple comparisons were made using the Bonferroni correction method. 95% confidence intervals (CIs) are reported for each estimated mean.

All reported p-values are bilateral. The level of significance was set at 5%. All statistical analyses were performed with R software v4.3.1.

## Results

### Patients’ characteristics

**Fig. 1:**
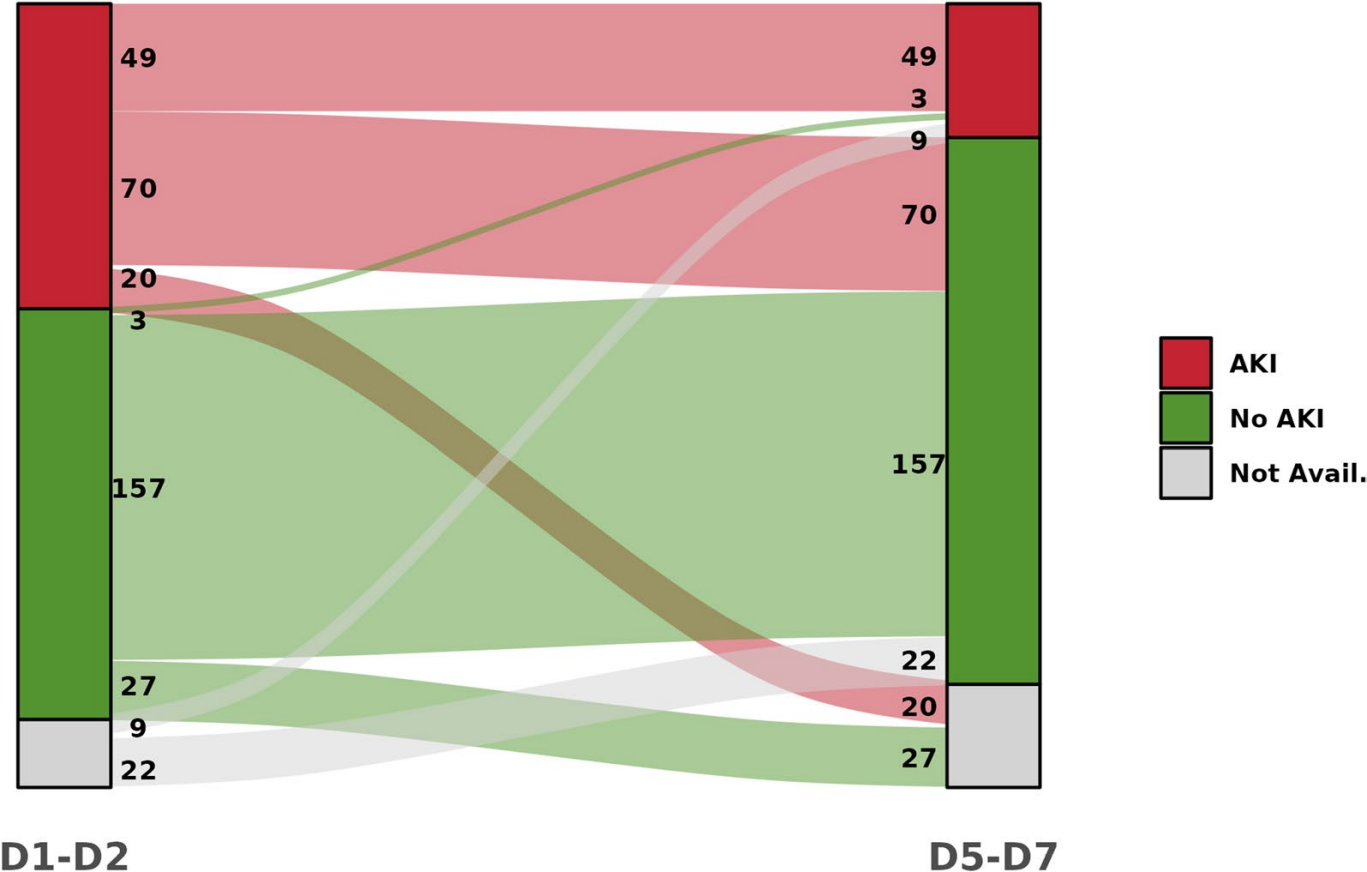
Alluvial plot representing the evolution of AKI status during the first week after injury. AKI: acute kidney injury. Not avail: Not available. Immunologic data were missing for 33 patients at D1-2 and 47 patients at D5-7, therefore these patients were removed from the analysis at the corresponding timepoint

Three hundred and fifty-nine patients were included in the REALAKI study. Among them 137 (38%) were trauma patients, 103 (29%) post-surgery patients, 95 (26%) sepsis patients, and 24 (7%) burn patients. The median age was 59 [44–70] years, the median SOFA score at admission was 4 [1–8], and the mortality rate at day 28 after admission was 5%. Upon admission, 139 (39%) patients presented with AKI, including 69 (19%) KDIGO stage 1, 30 (8%) KDIGO stage 2 and 40 (11%) KDIGO stage 3. At D5-7, 61 (20%) patients presented with AKI including 27 (9%) KDIGO stage 1, 10 (3%) KDIGO stage 2 and 24 (8%) KDIGO stage 3. Immunologic data were not available for 31 patients at D1-2 and for 47 patients at D5-7, therefore these patients were removed from the analysis at the corresponding timepoint. Among the 52 patients with AKI at D5-7 for whom immunologic data were available at D1-2, 49 (94%) presented with AKI at both D1-2 and D5-7 and therefore presented with persistent AKI (Fig. 1). Only 3 patients from the whole cohort presented with AKI at D5-7 while presenting no AKI at D3-4 or D1-2 and therefore could be defined as “late-onset AKI” (Additional figure S1). Overall, 39 (11%) patients required renal replacement therapy (RRT) at D1-2 while 28 (9%) patients required RRT at D5-7. Patients’ characteristics at D1-2 and D5-7 according to the presence of AKI are reported in Table 1 and Additional table S2 respectively.

**Table 1:** Patients’ characteristics at D1-2 in the AKI and no AKI groups

	No AKI (n = 187)	AKI (n = 139)	*p*-value
Demographics			
Sex, male, n (%)	110 (59)	104 (75)	0.004
Age, years	56 [41–67]	63 [49–75]	0.006
BMI	25 [22–27]	26 [23–29]	0.042
Injury, n (%)			
Trauma	88 (47)	48 (35)	< 0.001
Surgery	65 (35)	23 (17)	
Sepsis	22 (12)	60 (43)	
Burn	12 (6.4)	8 (5.8)	
Severity scores			
SAPSII	22 [17–30]	40 [29–51]	< 0.001
Charlson	0 [0–2]	1 [0–2]	0.157
SOFA	1 [1–5]	8 [5–10]	< 0.001
Biological values			
ALT (IU/L)	66 [34–158]	57 [25–152]	0.494
AST (IU/L)	76 [48- 152]	112 [49–218]	0.144
Bilirubin (mg/dL)	17 [10.3–35]	18 [12–32.5]	0.617
Creatinine (µmol/L)	69 [55–82]	130 [106–190]	< 0.001
Leucocytes (10^9^/L)	12 [10–16]	14 [11–18]	0.007
Lymphocytes (10^9^/L)	1.3 [0.9–1.8]	1.2 [0.8–1.8]	0.282
Monocytes (10^9^/L)	0.9 [0.6–1.2]	1 [0.6–1.5]	0.359
Neutrophils (10^9^/L)	9.5 [7.7–13]	12 [8.6–16]	0.002
Platelets (10^9^/L)	203 [164–258]	191 [136–244]	0.030
PaO2/FiO_2_	297 [191–392]	225[174–323]	0.031
Hemoglobin (g/L)	120 [105- 135]	112 [96–132]	0.032
pH	7.4 [7.3–7.4]	7.3 [7.2–7.4]	0.015
Lactate (mmol/L)	2 [1.3–2.9]	2.6 [1.9–3.7]	0.002
Organ failures			
Vasopressors, n (%)	70 (38)	110 (79)	< 0.001
Renal Replacement Therapy during ICU stay, n (%)	6 (3.2)	38 (27)	< 0.001
Invasive mechanical ventilation, n (%)	68 (36)	98 (71)	< 0.001
Outcomes			
Death at D28, n (%)	2 (1.1)]	14 (10)	< 0.001
ICU length of stay, days	5 [3–8]	8 [4.5–13.5]	< 0.001
Hospital-free days at D28	16 [7–22]	6 [0 – 16]	< 0.001

Results are expressed as medians (IQR) or n (%). Chi square or Fisher’s exact test were used for qualitative variables assessment. Quantitative variables were compared with Mann–Whitney U test or Student *t* tests according to the distribution of the variables

AKI: Acute kidney injury, ALT: alanine transaminase, AST: Aspartate transaminase, BMI: Body mass index, ICU: intensive care unit, SAPS: Simplified Acute Physiology Score, SOFA: Sequential Organ Failure Assessment

**Fig. 2:**
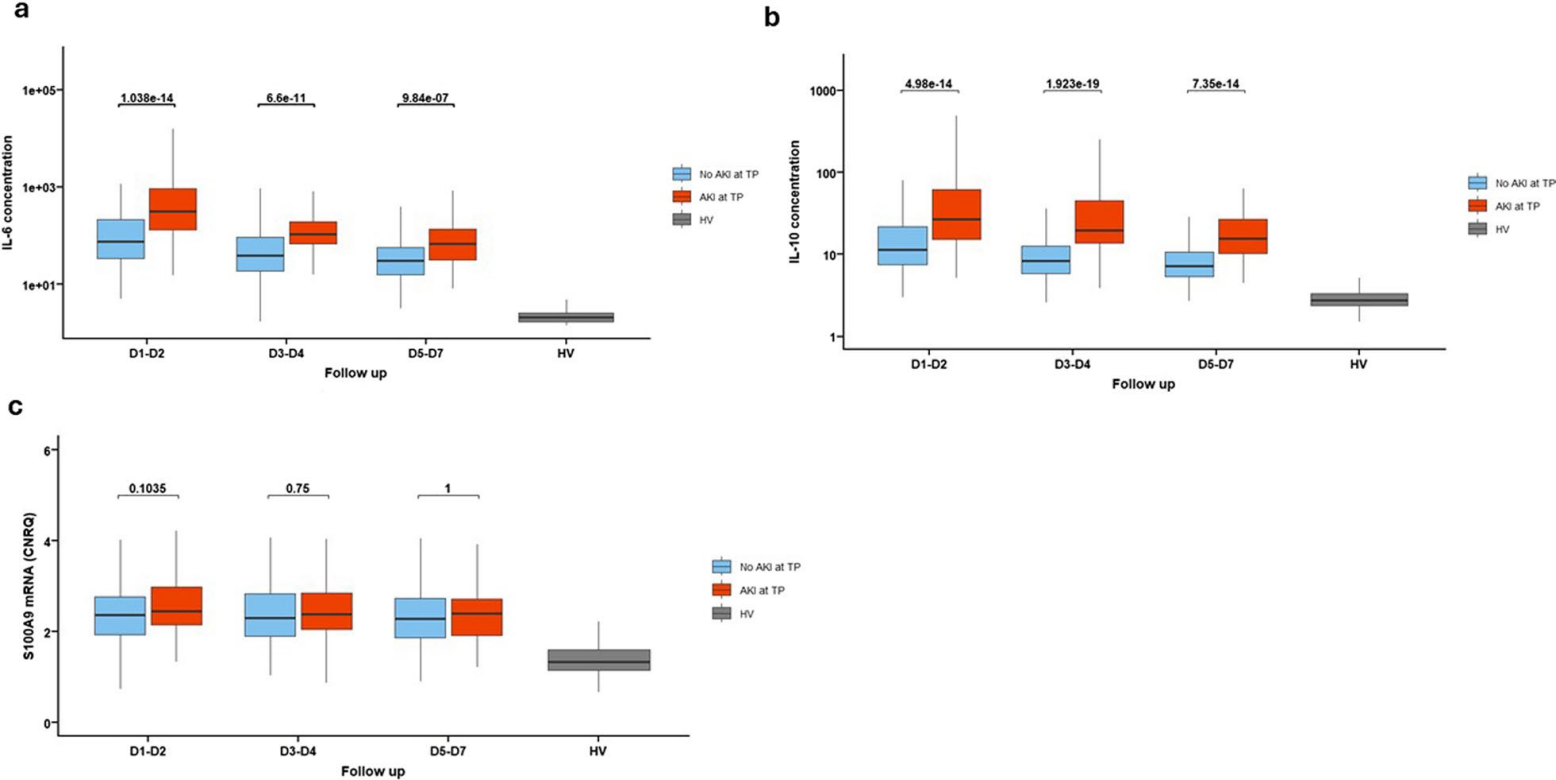
Evolution of inflammatory markers in the AKI and no AKI groups during the first week after injury. Classification in AKI and no AKI group is based on the presence of AKI at the corresponding time-point. **a** Interleukin-6 concentration (pg/mL). **b** Interleukin-10 concentration (pg/mL). **c** S100A9 alarmin messenger RNA expression. Results are presented as Tukey boxplots at each sampling time-point in each subgroup. Quantitative variables were compared with Mann–Whitney U test or Student t tests. Comparisons were adjusted for multiple comparisons using the Bonferroni method. AKI: acute kidney injury. IL-6 = Interleukin 6, IL-10 = Interleukin 10, HV: healthy volunteers, mRNA = messenger RNA, TP: time-point

A significant increase of both pro and anti-inflammatory cytokines was observed in the AKI group compared to the remaining cohort through the ICU time course. These alterations were more important at D1-2 and tended to decrease over time (Fig. 2a, b). However, the levels of S100A9 alarmin messenger RNA (mRNA) did not differ between AKI and non-AKI patients (Fig. 2c).

**Fig. 3:**
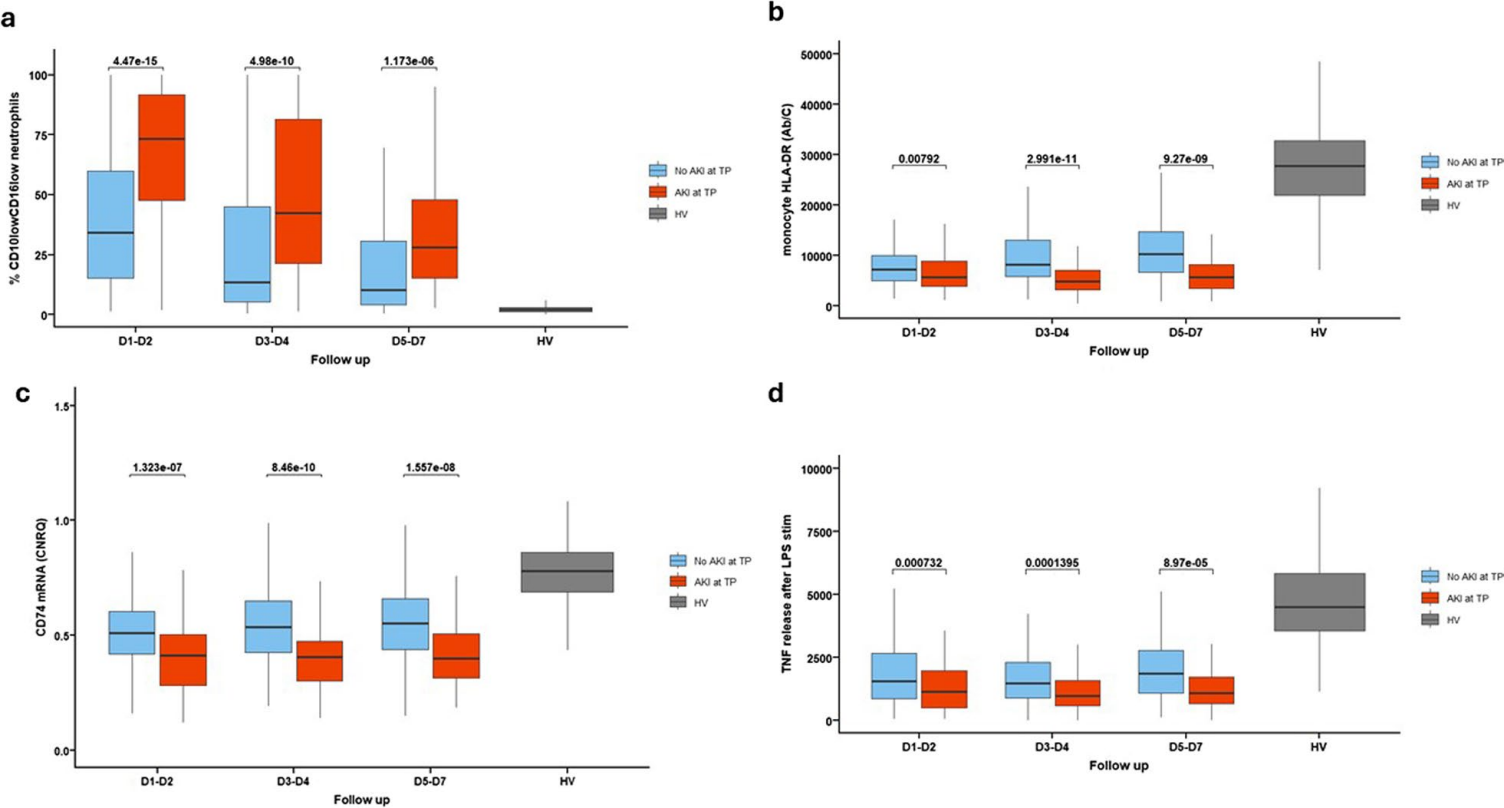
Evolution of innate immune response in the AKI and no AKI groups during the first week after injury. Classification in AKI and no AKI group is based on the presence of AKI at the corresponding time-point. **a** Percentage of CD10lowCD16low immature neutrophils. **b** monocyte HLA-DR (mHLA-DR) expression (Ab/C). **c** CD74 messenger RNA expression. **d** Ex vivo tumor necrosis factor-α (TNF-α) production in response to lipopolysaccharide. Results are presented as Tukey boxplots at each sampling time-point in each subgroup. Quantitative variables were compared with Mann–Whitney U test or Student t tests. Comparisons were adjusted for multiple comparisons using the Bonferroni method. Ab/C: number of anti-HLA-DR antibody bound per monocyte, AKI: acute kidney injury, HV: healthy volunteers, mRNA: messenger RNA, TNF-α: tumor necrosis factor-α, TP: time-point

Simultaneously, the innate immune response was significantly altered in AKI patients (Fig. 3). The proportion of CD10^low^CD16^low^ immature neutrophils was increased (Fig. 3a). The expression of monocyte Human Leucocyte Antigen DR (mHLA-DR) and of CD74 mRNA were both decreased (Fig. 3b, c). Circulating immune cells presented with an altered function characterized by a decrease in ex vivo tumor necrosis factor-α (TNF-α) production in response to lipopolysaccharide (LPS) (Fig. 3d). Similarly, CX3CR1 mRNA levels were lower in AKI patients. These changes in the innate immune response were present at D1-2 and persisted up to D5-D7 in patients with AKI.

**Fig. 4:**
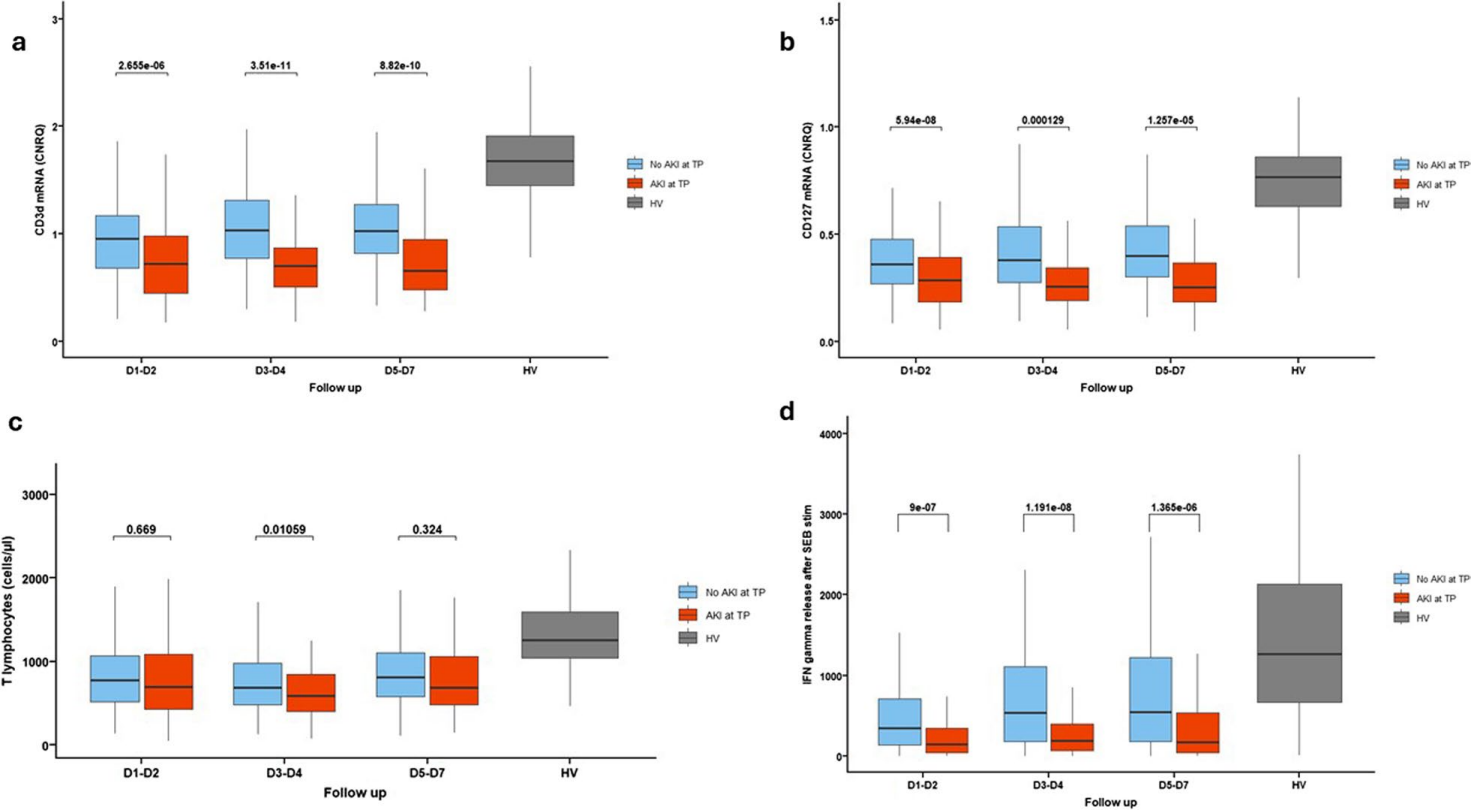
Evolution of adaptive immune response in the AKI and no AKI groups during the first week after injury. Classification in AKI and no AKI group is based on the presence of AKI at the corresponding time-point. **a** CD3D messenger RNA expression. **b** CD127 messenger RNA expression. **c** Interferon gamma release after SEB stimulation. Results are presented as Tukey boxplots at each sampling time-point in each subgroup. Quantitative variables were compared with Mann–Whitney U test or Student t tests. Comparisons were adjusted for multiple comparisons using the Bonferroni method. AKI: acute kidney injury, HV: healthy volunteers, IFN: Interferon, mRNA: messenger RNA, SEB: Staphylococcal Enterotoxin B, TP: time-point

Lastly, the adaptive immune response was also markedly impaired in the AKI group (Fig. 4). Quantitative alterations were observed as CD3D mRNA and CD127 mRNA levels were decreased during the first week (Fig. 4a, b). However, the absolute T lymphocyte count was not different between AKI and non-AKI patients (Fig. 4c). Concomitantly, circulating immune cells presented with functional alterations characterized by a decreased ex vivo interferon-γ production upon stimulation with Staphylococcal enterotoxin B (Fig. 4d). As observed with the innate immune response, the adaptive immune response tended to remain altered in the AKI groups as compared to patients with no AKI.

Importantly, the changes observed in the immune responses of AKI patients tended to be more pronounced with increasing KDIGO stages of AKI whether the modifications were related to inflammation or injury-induced immunosuppression (Additional figures S2, S3 and S4).

### Association of immune alterations with AKI in multivariable analysis

At D1-2, in the logistic regression model, an independent association was observed between AKI and CD10^low^CD16^low^ immature neutrophils (OR 3.0 [1.7–5.5]—p < 0.001) (Table 2). Among clinical parameters, age, sex, and modified SOFA score were associated with AKI whereas the type of injury was not.

**Table 2:** Association between AKI and immune parameters at D1-2 in multivariable analysis

	Odds ratio (95% CI)	*p*-value
Age, per year	1.02 [1.00 – 1.03]	0.02
Sex, male	2.33 [1.27 – 4.38]	0.007
Corrected SOFA score, per SOFA point	1.20 [1.10 – 1.30]	< 0.001
CD10^low^CD16^low^ immature neutrophils percentage	3.03 [1.70 – 5.50]	< 0.001

Logistic regression model at D1-2. Odds ratios calculated for immune parameters were normalized to an increment from first to third quartile to allow comparison between models. Only significant variables are reported in the table. Corrected SOFA score: SOFA score excluding the renal component. SOFA: Sequential Organ Failure Assessment

At D5-7, interleukin-10 (IL-10) levels and ex vivo TNF-α production after LPS stimulation were independently associated with the presence of AKI (OR 1.38 [1.12–1.71], *p* = 0.001 and 0.51 [0.27–0.91], *p* = 0.03 respectively). Age, sex, and modified SOFA score were also significantly associated with AKI in the model whereas the type of injury was not (Table 3).

**Table 3:** Association between AKI and immune parameters at D5-7 in multivariable analysis

	Odds ratio (95% CI)	p-value
Age, per year	1.03 [1.0–1.06]	0.01
Sex, male	2.86 [1.24–7.1]	0.02
Corrected SOFA score, per SOFA point	1.35 [1.15–1.58]	< 0.001
IL-10	1.38 [1.12–1.71]	0.001
TNF-α production after LPS stimulation	0.51 [0.27–0.91]	0.03

Logistic regression model at D5-7. Odds ratios calculated for immune parameters were normalized to an increment from first to third quartile to allow comparison between models. Only significant variables are reported in the table. Corrected SOFA score: SOFA score excluding the renal component. IL-10: Interleukin-10, LPS: Lipopolysaccharide, SOFA: Sequential Organ Failure Assessment, TNF- α: Tumor necrosis factor alpha

Furthermore, the presence of AKI at D5-D7 was associated with the initial pro and anti-inflammatory response as both IL-6 and IL-10 levels measured at D1-2 were independently associated with AKI at D5-7 (OR 1.12 [1.03–1.22], *p* = 0.009 and OR 1.25 [1.07–1.47], *p* = 0.004 respectively) after adjusting for age, modified SOFA score at D1-2 and presence of AKI at D1-2.

### Clinical and immune profile of patients presenting with AKI at D5-D7

Overall, the immune profile of patients with AKI at D5-7 was characterized by persistent inflammation and injury-induced immunosuppression (Additional table S3). In parallel, patients with AKI at D5-7 exhibited worse clinical outcomes. Clinical worsening, defined as the occurrence of death or nosocomial infection within the 30 days following ICU admission, was associated with AKI at D5-7 as 26 (44%) patients presented with clinical worsening in the AKI group versus 61 (25%) in the non-AKI group (*p* = 0.007). The sites of nosocomial infections diagnosed in AKI and non-AKI patients are reported in Additional Table S4.

Last, patients who recovered from AKI between D1-2 and D5-7 compared to patients with persistent AKI at D5-7, tended to correct these alterations, compared to patients with a persistent AKI at D5-7 (Table 4).

**Table 4:** Mixed model analysis of immunological parameters in patients with persistent AKI at D5-7 compared to patients correcting their AKI between D1-2 and D5-7

	Recovery from AKI (n = 70)	Persistent AKI (n = 49)	*p*-value
Log-transformed IL-6 (pg/mL)			
D1-2	5.6 [5.3–5.9]	6.4 [6–6.7]	0.01
D5-7	3.7 [3.4–4]	4.3 [3.9–4.7]	0.13
Log-transformed IL-10 (pg/mL)			
D1-2	3.2 [3–3.4]	4.0 [3.8–4.2]	< 0.001
D5-7	2.2 [2–2.4]	2.8 [2.6–3]	< 0.001
S100A9 mRNA (cnrq_taq)			
D1-2	2.7 [2.5–2.8]	2.7 [2.5–2.9]	1
D5-7	2.4 [2.2 -2.6]	2.5 [2.2–2.7]	1
% CD10lowCD16low immature neutrophils			
D1-2	62 [55–68]	75 [68–83]	0.05
D5-7	24 [15–30]	38 [30–45]	0.04
CD74 mRNA (cnrq_taq)			
D1-2	0.45 [0.41–0.49]	0.35 [0.31–0.4]	0.004
D5-7	0.51 [0.5–0.6]	0.41 [0.36–0.45]	< 0.001
mHLA-DR (ab/cell)			
D1-2	7671 [6247–9095]	7421 [5704–9138]	1
D5-7	9362 [7930–10795]	6390 [4688–8092]	0.05
CX3CR1 mRNA expression (cnrq_taq)			
D1-2	1.17 [1.04–1.29]	0.84 [0.7–0.99]	0.006
D5-7	1.32 [1.26–1.5]	1.11 [0.97–1.26]	0.04
TNF-α production after LPS stimulation (pg/mL)			
D1-2	1662 [1350–1974]	1018 [645–1391]	0.06
D5-7	2084 [1770–2398]	1337 [964–1709]	0.02
T cells (n/µL)			
D1-2	868 [747–989]	770 [669–992]	1
D5-7	870 [749–991]	845 [699–992]	1
CD3D mRNA expression (cnrq_taq)			
D1-2	0.89 [0.8–0.97]	0.65 [0.55–075]	0.002
D5-7	0.99 [0.91–1.08]	0.76 [0.66–0.86]	0.002
CD127 mRNA expression (cnrq_taq)			
D1-2	0.36 [0.32–0.39]	0.26 [0.22–0.31]	0.01
D5-7	0.4 [0.36–0.44]	0.29 [0.25–0.34]	0.002
IFN-γ production after SEB stimulation (pg/mL)			
D1-2	350 [243–457]	169 [42–297]	0.19
D5-7	649 [542–756]	318 [191–446]	< 0.001

Linear mixed model (LMM) accounting for the interaction between time-points and the AKI status at D5-7 (recovery from AKI or persistent AKI), incorporating random effects for patients to handle intra-subject correlation of repeated measurements. IL-6 and IL-10 data were log-transformed prior to modeling. Pairwise comparison tests were performed between groups and timepoints using estimated marginal means derived from the LMM. Adjustments for multiple comparisons were made using the Bonferroni correction method. 95% confidence intervals (CIs) are reported for each estimated mean.

IL-6: Interleukin-6, IL-10: Interleukin-10, IFN: Interferon, LPS: lipopolysaccharide, mHLA-DR: monocyte Human Leucocyte Antigen DR, mRNA: messenger RNA, SEB: Staphylococcal Enterotoxin B, TNF: Tumor necrosis factor

## Discussion

Our study reports a significant increase in inflammatory markers as well as alterations in the innate and adaptive immune responses in AKI patients following different types of injuries. These alterations were proportional to AKI severity. Particularly, we found an independent association between an inflammatory profile and AKI at the early phase after the initial injury whereas markers of immunosuppression were associated with AKI at the end of the first week post initial injury. Importantly, patients with persistent AKI failed to restore an immune homeostasis compared to patients who recovered from AKI during the first week or compared to patients with no AKI.

In animal models, a large array of inflammatory markers and cell types is known to be associated with AKI. In AKI patients, several investigators have reported higher circulating plasma concentrations of inflammatory mediators [5, 6, 11]. In addition, early inflammation in septic shock patients—as exhibited by elevated levels of pro-inflammatory cytokines—predicts poor outcomes in AKI patients [12]. Although it is nowadays clear that AKI is associated with systemic inflammation, most studies did not report the evolution of different biomarkers during the first week and their association with AKI [10]. In the present study, we show a sustained increase of inflammatory markers during the first week in patients with AKI.

Importantly, our study highlights the association between AKI and a profile of injury-induced immunosuppression. Several reports have found that neutrophil recruitment into inflamed organs decreased significantly during AKI, thus reducing bacterial killing [21–23]. Besides, impaired monocyte function has also been described in patients with AKI and occurs contemporaneously with elevated plasma levels of proinflammatory cytokines [24]. However, all these studies included a low number of patients, focused on one aspect of immunosuppression and on biomarkers measured sporadically. We report herein a broad overview of the injury-induced immunosuppression profile presented by patients with AKI. This profile was more marked in patients with AKI at D5-7, who presented with quantitative (higher plasmatic IL-10 concentration, higher percentage of immature CD10l^ow^CD16^low^ immature neutrophils, lower monocyte Human Leucocyte Antigen-DR expression, lower CD3D and CD127 mRNA expressions) and qualitative (lower TNF-α production after LPS stimulation and lower IFN-γ production after SEB stimulation) alterations of their immune system.

An independent correlation was found between AKI at D5-7 and high plasmatic IL-10 concentration and low TNF-α production after LPS stimulation. As previously said, AKI is a risk factor for the development of sepsis in numerous clinical situations [8, 11, 25, 26]. However, most of the available findings to date only described the association between AKI and infections. Our findings are in line with these observations and describe AKI at D5-7 after injury, as an immunosuppressive state that could explain this increased risk of sepsis. We also report a higher risk of nosocomial infections or death in patients with AKI. These unfavorable outcomes are known to be associated with injury-induced immunosuppression [7]. Lastly, the type of injury in our study (sepsis, surgery, trauma or burns) did not modify the association between AKI and the alterations of the immune system observed. Accordingly, a profile of injury induced-immunosuppression has been described in these different clinical settings [27–29].

The immunosuppressive profile of patients with AKI at D5-7 can also be explained by the persistent inflammation measured at D5-7. The presence of low-grade inflammation and cellular activation may sustain the process of injury-induced immunosuppression [30, 31]. In addition, the intensity of the initial pro-inflammatory response could account for the delayed increased levels of circulating IL-10 as pro-inflammatory cytokines may upregulate the production of IL-10 [32].

To date, two main processes have been proposed to explain how AKI may alter the immune system [33]. First, a dysregulation of cytokine homeostasis in these patients may lead to increased inflammation. Conversely, an anti-inflammatory state exacerbated in AKI patients could result in clinical worsening or new infections. Our results support these two nonexclusive theories and emphasize the importance of AKI associated immunosuppression, especially in patients with persistent AKI.

The REALAKI study has several limitations. First, only creatinine levels were used to classify AKI patients according to the KDIGO classification. As the REALISM study was not designed to specifically address kidney function, the urine output information was not available for all patients at all time-points, thus we chose to use solely the creatinine component of the KDIGO classification to limit measurement bias in urine output. Undoubtedly, the use of both oliguria and increased serum creatinine better defines AKI and patients with both criteria present exhibit worse outcomes than patients with one criterion [34]. Nevertheless, the isolated use of creatinine may still allow to determine patients with decreased chance of renal recovery. Second, the study was a single-center study, however patients were admitted to three different specialized ICUs (trauma center, burn center and medico-surgical ICU) allowing us to identify AKI after different types of injuries. Third, the overall severity in our cohort was low since patients after elective surgery or trauma were included. Our results confirm that AKI is associated with immune alterations even in less severe patients. Last, only plasmatic markers were evaluated in the present study so that direct mechanisms occurring within the kidney itself were not studied. However, the main purpose of the REALAKI was precisely to characterize the general immune profile of AKI patients to depict kidney-immune system crosstalks.

## Conclusion

Following various types of severe injuries including sepsis, surgery, trauma, and burns, early AKI is independently associated with the importance of the initial inflammatory response. Presence of AKI at one week after injury is associated with injury-induced immunosuppression. Patients who recover from AKI between the initial injury and the end of the first week tend to correct these immune alterations compared to patients with persistent AKI. A better understanding of the immune profile of AKI patients may help develop specific strategies to improve kidney function recovery and reduce AKI-related complications such as sepsis.

### Supplementary Information

**Additional file 1**. Immune parameters included in the study and significance of the values reported.

**Additional file 2**. CONSORT Diagram

**Additional file 3**. Evolution of inflammatory markers depending on KDIGO stages during the first week after injury. Classification in KDIGO stages is based on the KDIGO stage at the corresponding time-point. a. Interleukin-6 concentration (pg/mL). b. Interleukin-10 concentration (pg/mL). c.S100A9 alarmin messenger RNA. Results are presented as Tukey boxplots at each sampling time-point in each subgroup. IL-6: Interleukin 6, IL-10: Interleukin 10, mRNA: messenger RNA.

**Additional file 4**. Evolution of innate immune response depending on KDIGO stages during the first week after injury. Classification in KDIGO stages is based on the KDIGO stage at the corresponding time-point. a. Percentage of CD10lowCD16low immature neutrophils. b. monocyte HLA-DR (mHLA-DR) expression (Ab/C). c. CD74 messenger RNA expression. d. Ex vivo tumor necrosis factor-α (TNF-α) production in response to lipopolysaccharide (LPS) (pg/mL). Results are presented as Tukey boxplots at each sampling time-point in each subgroup. Ab/C: number of anti-HLA-DR antibody bound per monocyte, mRNA: messenger RNA, TNF-α: tumor necrosis factor-α.

**Additional file 5**. Evolution of adaptive immune response depending on KDIGO stages during the first week after injury. Classification in KDIGO stages is based on the KDIGO stage at the corresponding time-point. a. CD3D messenger RNA expression. b. CD127 messenger RNA expression. c. Interferon gamma release after SEB stimulation. Results are presented as Tukey boxplots at each sampling time-point in each subgroup. IFN: Interferon, mRNA: messenger RNA, SEB: Staphylococcal Enterotoxin B.

## Data Availability

The study datasets are available from the corresponding author on reasonable request.

## Abbreviations

AKI: Acute kidney injury
HAI: Hospital acquired infection
ICU: Intensive care unit
IFN-γ: Interferon gamma
IL-6: Interleukin-6
IL-10: Interleukin-10
KDIGO: Kidney Disease Improving Global Outcomes
LPS: Lipopolysaccharide
mHLA-DR: Monocyte Human Leucocyte Antigen DR
mRNA: Messenger RNA
SOFA: Sequential Organ Failure Assessment
TNF-α: Tumor necrosis factor-α
