# Supplementary material for: Immune profiling of critically ill patients with acute kidney injury during the first week after various types of injuries: the REALAKI study
Source: Crit Care. 2024 Jul 8;28:227. doi: 10.1186/s13054-024-04998-w (PMC11232205; doi:10.1186/s13054-024-04998-w)
Supplement: Supplementary file 1 — Additional file 1. Immune parameters included in the study and significance of the values reported. [file 13054_2024_4998_MOESM1_ESM.docx]

**Additional table 1: Immune parameters included in the study and significance of the values reported**

| **Inflammatory markers** |  |
| --- | --- |
| IL-6 | Pro-inflammatory cytokine |
| IL-10 | Anti-inflammatory cytokine |
| S100A9 mRNA expression | S100A9 is a Ca2+ binding protein belonging to the S100 family, modulates the inflammatory response by stimulating leukocyte recruitment and inducing cytokine secretion |
| **Innate immune response** |  |
| % CD10lowCD16low immature neutrophils | Immature neutrophils associated with the inflammatory response at the early phase after injury |
| CD74 mRNA expression | CD74 is the HLA-DR antigen-associated invariant chain, decreased in case of injury-induced immunosuppression |
| mHLA-DR | Major histocompatibility complex (MHC) class II–mediated antigen-presentation, decreased in case of injury-induced immunosuppression |
| CX3CR1 mRNA expression | CX3XR1 is the receptor of CX3CR1 ligand (fractalkine), expressed on some leucocyte subpopulations especially monocytes, decreased in case of injury-induced immunosuppression |
| TNF-α production after LPS stimulation | Functional assay to assess monocyte function, decreased in case of injury-induced immunosuppression |
| **Adaptive immune response** |  |
| CD3D mRNA expression | CD3D is the T-cell surface glycoprotein CD3 delta chain, part of the T-cell receptor. Role in signal transduction in T cell activation. Decreased in case of injury-induced immunosuppression |
| CD127 mRNA expression | CD127 is the interleukin-7 receptor subunit alpha expressed in early thymocytes, T cells, pre-B cell. Role in regulating the development and homeostasis of T-cells. Decreased in case of injury-induced immunosuppression |
| IFN-γ production after SEB stimulation | Functional assay to assess lymphocyte function, decreased in case of injury-induced immunosuppression |

*IL-6: Interleukin-6, IL-10: Interleukin-10, IFN: Interferon, LPS: lipopolysaccharide, mHLA-DR: monocyte Human Leucocyte Antigen DR, mRNA: messenger RNA, SEB: Staphylococcal Enterotoxin B, TNF: Tumor necrosis factor.*

**Additional table 2. Patients’ characteristics at D5-7 in the AKI and no AKI groups**

|  | **No AKI**  **(n = 249)** | **AKI**  **(n=61)** | **p-value** |
| --- | --- | --- | --- |
| **Demographics** |  |  |  |
| Sex, male, n (%) | 162 (65) | 45 (74) | 0.253 |
| Age, years | 57 [43- 70] | 68 [59 - 80] | <0.001 |
| BMI, kg/m^2^ | 25 [22 - 28] | 27 [24 - 30] | 0.001 |
| **Injury, n (%)** |  |  |  |
| Trauma  Surgery  Sepsis  Burn | 112 (450)  75 (30)  44 (18)  18 (7.2) | 7 (12)  10 (16)  39 (64)  5 (8.2) | <0.001 |
| **Severity scores** |  |  |  |
| SAPSII | 25 [19 - 37] | 47 [35 - 57] | <0.001 |
| Charlson | 0 [0 - 2] | 2 [0 - 3] | 0.001 |
| SOFA at D5-7 | 1 [0 - 1] | 4 [2 - 7] | <0.001 |
| **Biological values** |  |  |  |
| ALT (IU/L) | 48 [30 - 94] | 77 [17 - 219] | 0.616 |
| AST (IU/L) | 44 [33 - 67] | 114 [34 - 218] | 0.014 |
| Bilirubin (mg/dL) | 10 [7 - 20] | 37 [14 - 71] | 0.004 |
| Creatinine (µmol/L) | 59 [48 - 74] | 123 [99 - 207] | <0.001 |
| Leucocytes (10^9^/L) | 9.2 [7.4 - 12] | 12 [8.7 - 16.02] | <0.001 |
| Lymphocytes (10^9^/L) | 1.3 [0.9 - 1.6] | 1.2 [0.7 - 1.8] | 0.531 |
| Monocytes (10^9^/L) | 0.8 [0.6 - 1.1] | 0.9 [0.6 - 1.4] | 0.158 |
| Neutrophils (10^9^/L) | 6.7 [5 - 8.9] | 9.5 [6.2 - 12] | <0.001 |
| Platelets (10^9^/L) | 250 [190 - 331] | 203 [113 - 310] | 0.002 |
| PaO2/FiO2 | 262 [187 - 332] | 216 [145 - 298] | 0.266 |
| Hemoglobin (g/L) | 104 [92 - 119] | 94 [85 - 106] | 0.001 |
| pH | 7.4 [7.4 - 7.5] | 7.2 [7.3 - 7.5] | 0.438 |
| Lactate (mmol/L) | 1.4 [1.1 - 1.7] | 1.8 [1.3 - 2.6] | 0.057 |
| **Organ failures** |  |  |  |
| Vasopressors, n (%) | 119 (48) | 57 (94) | <0.001 |
| Invasive mechanical ventilation, n (%) | 110 (44) | 50 (82) | <0.001 |
| **Outcomes** |  |  |  |
| Death at D28 | 1 (0.4) | 7 (112) | <0.001 |
| Clinical worsening (healthcare associated infection or death) at D28 | 61 (25) | 26 (43) | 0.007 |
| ICU length of stay, days | 6 [4 - 9] | 12 [8 - 21] | <0.001 |
| Hospital-free days at D28 | 15 [4 - 21] | 0 [0 - 6.5] | <0.001 |

**Additional table 3. Immune profile at D5-7 of patients with and without AKI at D5-7**

|  | **No AKI (n = 249)** | **AKI (n = 61)** | **p-value** |
| --- | --- | --- | --- |
| **IL-6 (pg/mL)** | 30 [16 - 57] | 68 [32 - 136] | <0.001 |
| **IL-10 (pg/mL)** | 7.2 [5.4 - 11] | 16 [10 - 27] | <0.001 |
| **S100A9 mRNA expression (cnrq_taq)** | 2.3 [1.9 - 2.8] | 2.4 [2 - 2.7] | 0.391 |
| **% CD10lowCD16low immature neutrophils** | 10 [4.2 - 31] | 28 [15 - 48] | <0.001 |
| **CD74 mRNA expression (cnrq_taq)** | 0.6 [0.4 - 0.7] | 0.4 [0.3 - 0.5] | <0.001 |
| **mHLA-DR (ab/cell)** | 10254 [6735 - 14742] | 5685 [3518 - 8239] | <0.001 |
| **CX3CR1 mRNA (cnrq_taq)** | 1.4 [1.2 - 1.8] | 1.1 [0.7 - 1.4] | <0.001 |
| **TNF-α production after LPS stimulation (pg/mL)** | 1865 [1098 - 2782] | 1099 [663 - 1736] | <0.001 |
| **T cells (n/µL)** | 808 [582 - 1101] | 690 [483 - 1056] | 0.108 |
| **CD3D mRNA expression**  **(cnrq_taq)** | 1 [0.8 - 1.3] | 0.7 [0.5 - 1] | <0.001 |
| **CD127 mRNA expression**  **(cnrq_taq)** | 0.4 [0.3 - 0.5] | 0.3 [0.2 - 0.4] | <0.001 |
| **IFN-γ production after SEB stimulation**  **(pg/mL)** | 549 [187 - 1223] | 179 [44 - 538] | <0.001 |

**Additional table 4.** **Incidence and sites of nosocomial infections until D30 in AKI and non-AKI patients at D5-7**

|  | **No AKI (n = 245)** | **AKI (n= 59)** |
| --- | --- | --- |
| **Pneumonia** | 18 (7%) | 8 (13%) |
| **Intra-abdominal** | 10 (4%) | 4 (7%) |
| **Urinary tract** | 18 (7%) | 2 (3%) |
| **Other** | 14 (6%) | 8 (13%) |
