# Supplementary material for: Immune profiling of critically ill patients with acute kidney injury during the first week after various types of injuries: the REALAKI study
Source: Crit Care. 2024 Jul 8;28:227. doi: 10.1186/s13054-024-04998-w (PMC11232205; doi:10.1186/s13054-024-04998-w)
Supplement: Supplementary file 2 — Additional file 2. CONSORT Diagram [file 13054_2024_4998_MOESM2_ESM.pptx]

## Slide 1
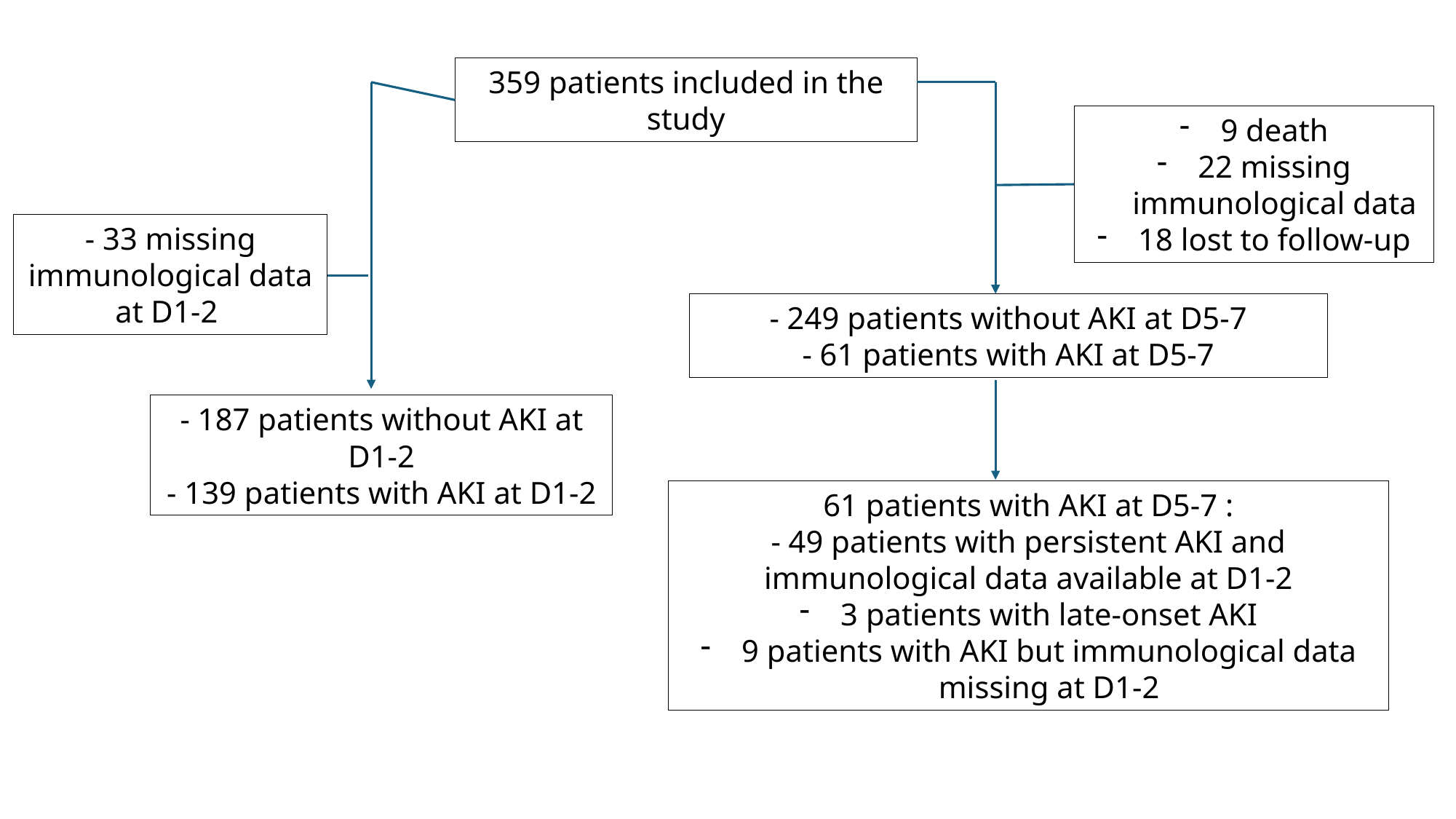

359 patients included in the study
9 death
22 missing immunological data
18 lost to follow-up
- 33 missing immunological data at D1-2
- 249 patients without AKI at D5-7
- 61 patients with AKI at D5-7
- 187 patients without AKI at D1-2
- 139 patients with AKI at D1-2
61 patients with AKI at D5-7 :
- 49 patients with persistent AKI and immunological data available at D1-2
3 patients with late-onset AKI
9 patients with AKI but immunological data missing at D1-2
