# Supplementary figures and images for: Immune profiling of critically ill patients with acute kidney injury during the first week after various types of injuries: the REALAKI study
Source: Crit Care. 2024 Jul 8;28:227. doi: 10.1186/s13054-024-04998-w (PMC11232205; doi:10.1186/s13054-024-04998-w)

## Slide 1
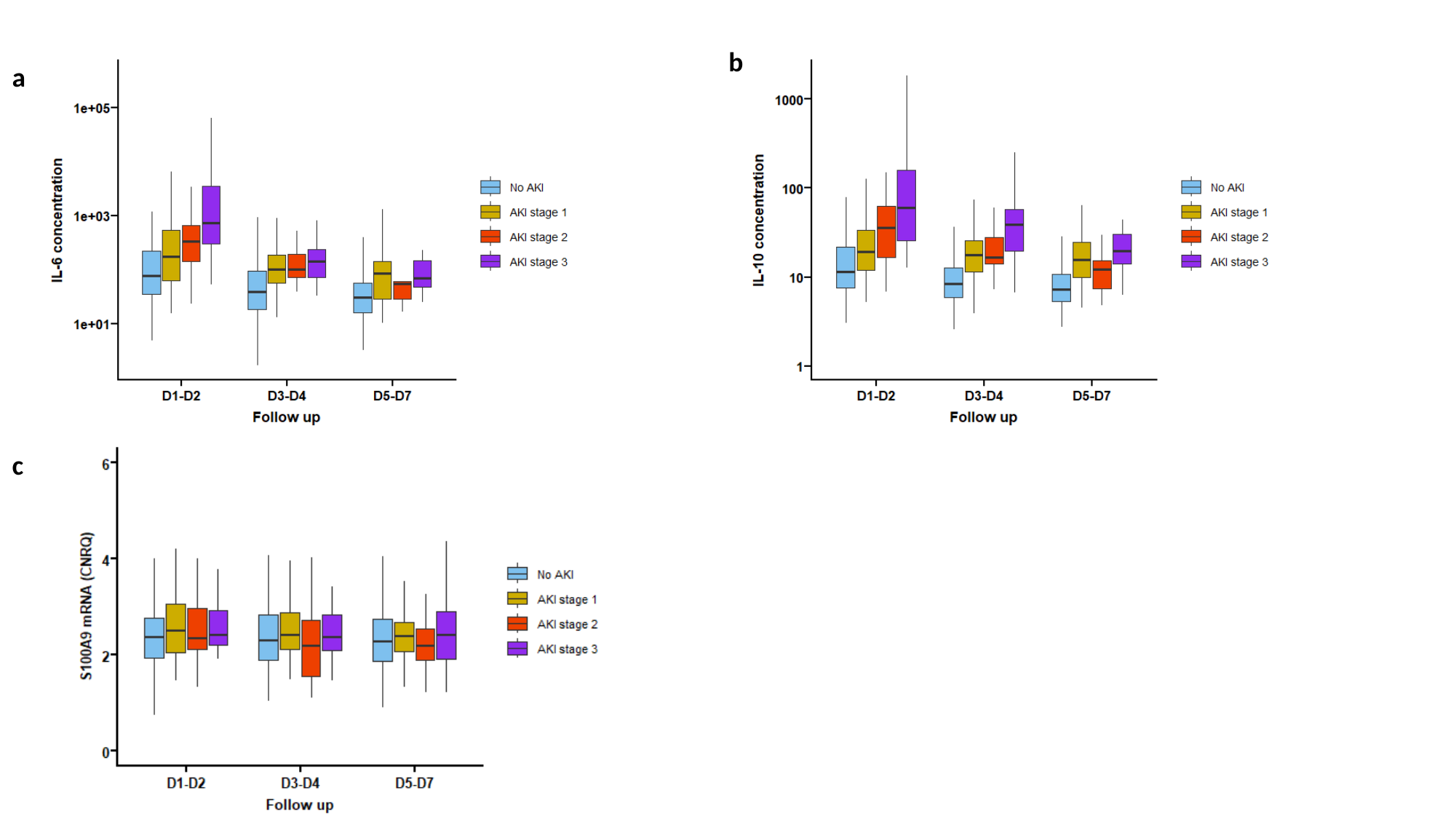

b
a
c

Supplement: Supplementary file 3 — Additional file 3. Evolution of inflammatory markers depending on KDIGO stages during the first week after injury. Classification in KDIGO stages is based on the KDIGO stage at the corresponding time-point. a. Interleukin-6 concentration (pg/mL). b. Interleukin-10 concentration (pg/mL). c.S100A9 alarmin messenger RNA. Results are presented as Tukey boxplots at each sampling time-point in each subgroup. IL-6: Interleukin 6, IL-10: Interleukin 10, mRNA: messenger RNA. [file 13054_2024_4998_MOESM3_ESM.pptx]

## Slide 1
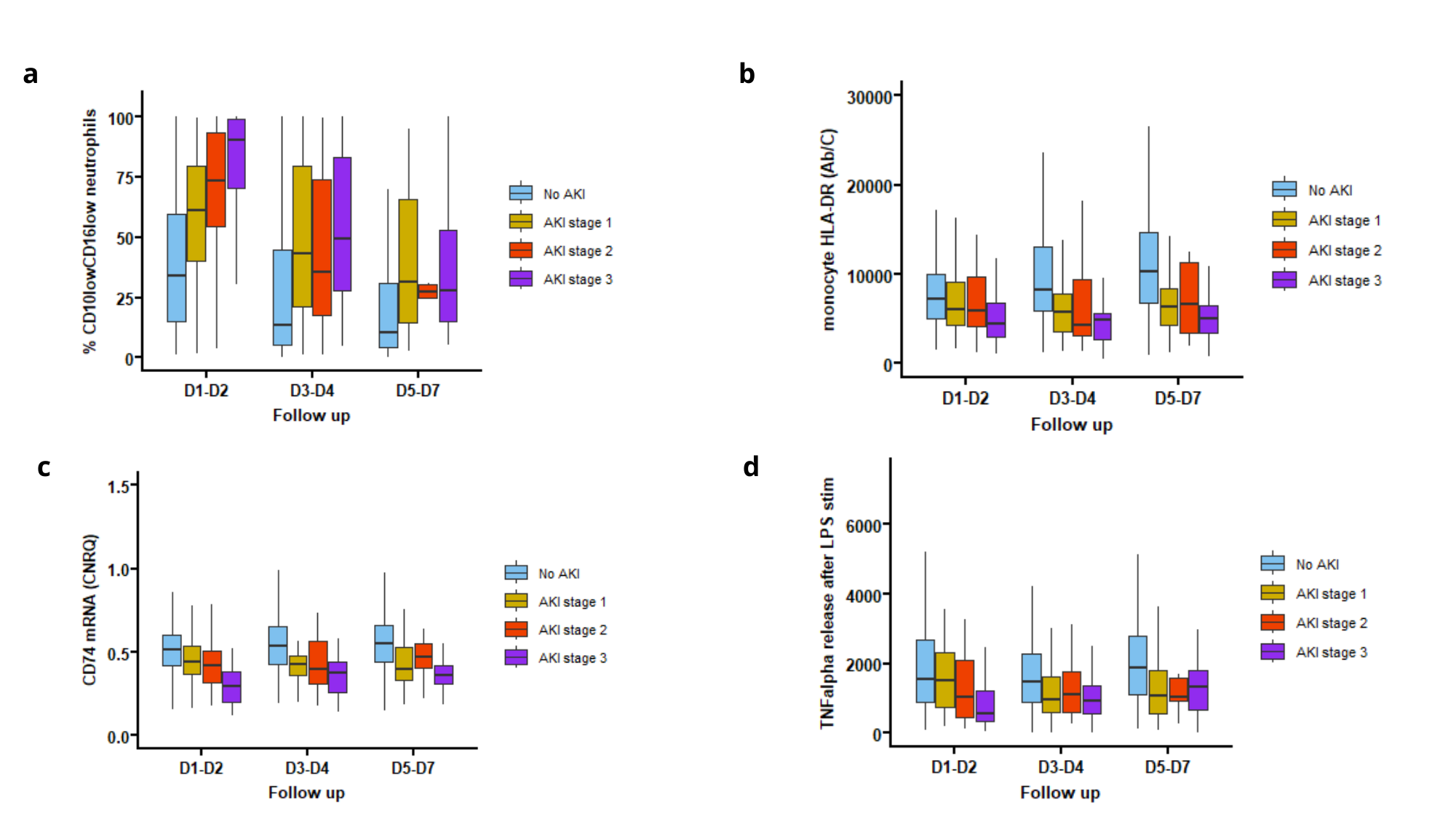

a
b
c
d

Supplement: Supplementary file 4 — Additional file 4. Evolution of innate immune response depending on KDIGO stages during the first week after injury. Classification in KDIGO stages is based on the KDIGO stage at the corresponding time-point. a. Percentage of CD10lowCD16low immature neutrophils. b. monocyte HLA-DR (mHLA-DR) expression (Ab/C). c. CD74 messenger RNA expression. d. Ex vivo tumor necrosis factor-α (TNF-α) production in response to lipopolysaccharide (LPS) (pg/mL). Results are presented as Tukey boxplots at each sampling time-point in each subgroup. Ab/C: number of anti-HLA-DR antibody bound per monocyte, mRNA: messenger RNA, TNF-α: tumor necrosis factor-α. [file 13054_2024_4998_MOESM4_ESM.pptx]

## Slide 1
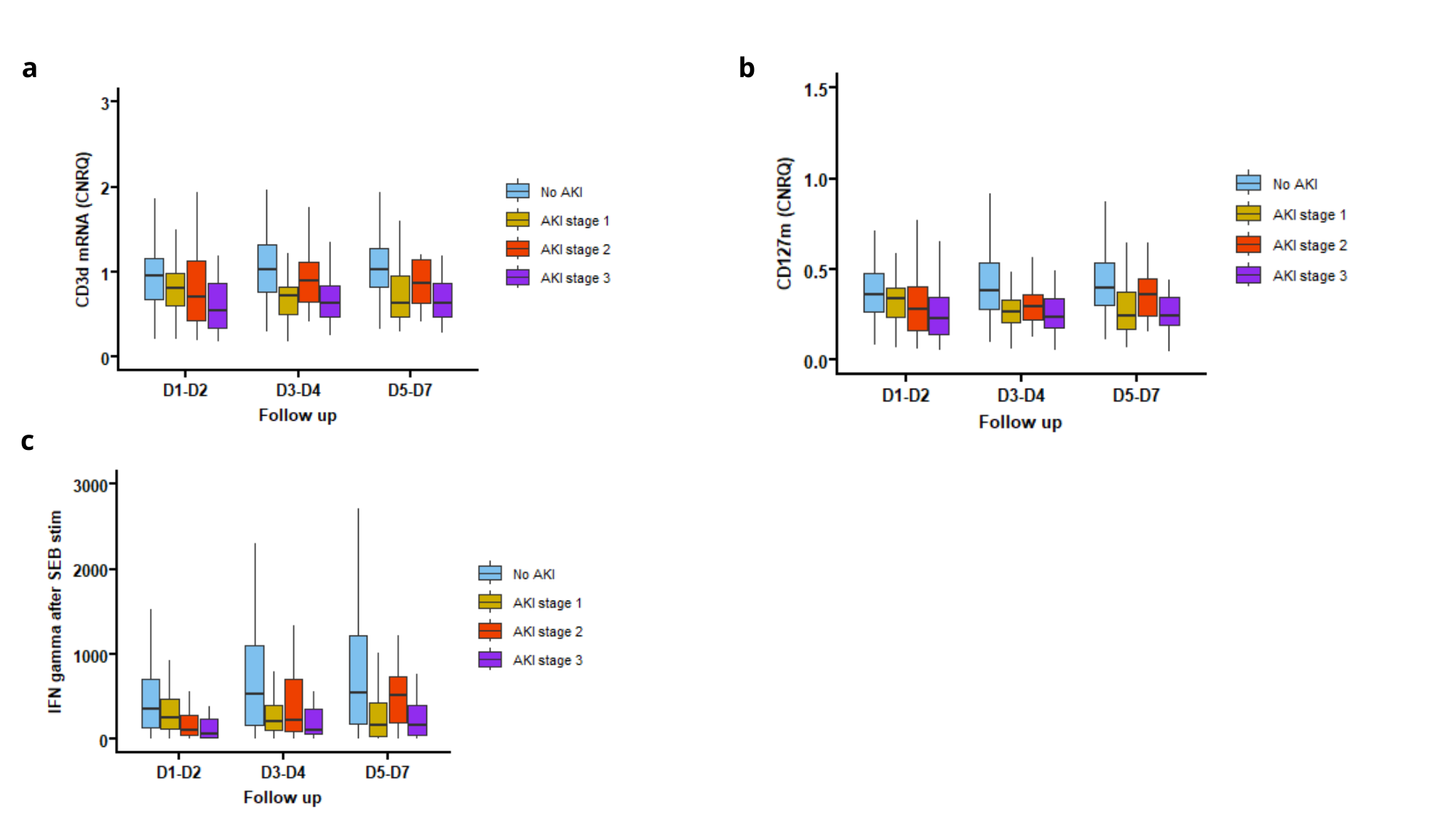

a
b
c

Supplement: Supplementary file 5 — Additional file 5. Evolution of adaptive immune response depending on KDIGO stages during the first week after injury. Classification in KDIGO stages is based on the KDIGO stage at the corresponding time-point. a. CD3D messenger RNA expression. b. CD127 messenger RNA expression. c. Interferon gamma release after SEB stimulation. Results are presented as Tukey boxplots at each sampling time-point in each subgroup. IFN: Interferon, mRNA: messenger RNA, SEB: Staphylococcal Enterotoxin B. [file 13054_2024_4998_MOESM5_ESM.pptx]
